# United States Veterinary Medical Association

**Published:** 1892-03

**Authors:** 


The following appointments have been made:

*Committee of Arrangements for the Annual Meeting at Boston, 1892:* W. H. Hoskins, Secretary, 12 South 37th street; J. F. Winchester, L. H. Howard.

*Committee of Arrangements for the Annual and International Meeting at Chicago, 1893:* R. S. Huidekoper, M. D., Veterinarian, (Alfort) President, 311 West 59th street, N. Y.; W. L. Williams, V. S., Vice-President, Purdue University, Lafayette, Ind.; W. H. Hoskins, D. V. S., Secretary, 12 South 37th street, Philadelphia; D. E. Salmon, Chief of the Bureau of Animal Industry, Washington, D. C.; A. Liautard, M. D., V. M., American Veterinary College, New York; A. H. Baker, V. S., Chicago Veterinary College, Chicago; Olof Schwartzkopff, V. M. D., University of Minnesota, Veterinary Department, Minneapolis, Minn.; J. H. Stickney, M. R. C. V. S., Boston, Mass.; A. W. Clement, V. S., Johns Hopkins University, Baltimore, Md.

